# Bioleptin as a useful marker of metabolic status in children with diabetes mellitus type 1

**DOI:** 10.3389/fendo.2023.1235409

**Published:** 2023-08-21

**Authors:** Katarzyna Jakubek-Kipa, Sabina Galiniak, Katarzyna Łagowska, Izabela Krupa, Aleksandra Ludwin, Jacek Tabarkiewicz, Artur Mazur

**Affiliations:** ^1^ Department of Pediatrics, Institute of Medical Sciences, Medical College of Rzeszów University, Rzeszów, Poland; ^2^ Department of Biochemistry and General Chemistry, Institute of Medical Sciences, Medical College of Rzeszów University, Rzeszów, Poland; ^3^ Laboratory for Translational Research in Medicine, Centre for Innovative Research in Medical and Natural Sciences, Medical College of Rzeszów University, Rzeszow, Poland; ^4^ Department of Human Immunology, Institute of Medical Sciences, Medical College of Rzeszów University, Rzeszów, Poland

**Keywords:** leptin, bioleptin, diabetes, children, BMI

## Abstract

**Introduction:**

The purpose of our study was tomeasure the level of leptin and biologically active leptin (bioLEP) in children with type 1 diabetes, depending on the duration of diabetes and its degree of metabolic control.

**Methods:**

The study included 94 children (58 boys and 36 girls). In a group of children with diabetes, 40 patients were newly diagnosed with type 1 diabetes, 40 children who have diabetes for more than a year (20 with good metabolic control and 20 with poor metabolic control). The control group consisted of 14 healthy children. The serum level of leptin and bioLEP was measured using a sandwich enzyme-linked immunosorbent assay. To our knowledge, this is the first study to describe bioLEP levels among diabetic children with different forms of disease control.

**Results:**

Lower levels of leptin were found in children with diabetes compared to healthy children. Furthermore, we found a statistically higher concentration of leptin in the group of children with newly diagnosed diabetes compared to children from the diabetic group with poor metabolic control and lower than healthy children (11.19 vs. 7.84 and 20.94 ng/mL). Moreover, children in the metabolically well-controlled group had statistically lower levels of this hormone (5.11 ng/mL) than healthy children. Leptin concentrations differed significantly between underweight, overweight, and obese children.

**Discussion:**

In our study, the level of bioLEP differed significantly between children in the newly diagnosed diabetes group and children in the long-term, poorly controlled diabetes group and healthy controls. Despite many studies published in recent years, many aspects of leptin secretion, action, and mechanisms of its influence on carbohydrate and fat metabolism are still to be clarified. In our opinion, studies evaluating the status of bioLEP in diabetes can also contribute to a better understanding of the mechanisms regulating metabolism.

## 1 Introduction

Type 1 and type 2 diabetes of youth is a major problem around the world. Reports suggest that among all cases of diabetes diagnosed in the US type 1 and type 2 diabetes account for approximately 6% and 91%, respectively (1, 2). Leptin, a 16 kDa protein hormone secreted into the circulation from white adipocytes, has been implicated in the regulation of food intake, body mass, and reproductive function and plays a role in fetal growth, pro-inflammatory immune responses, angiogenesis, and lipolysis (3, 4). Recent evidence suggests that the adiposity hormone leptin also plays an important role in controlling glucose metabolism through its actions in the brain (5). Circulating leptin is secreted into the bloodstream, reaches the brain through the blood-brain barrier, and acts on the hypothalamus (6). Both *in vivo* and *in vitro* studies suggest that insulin stimulates leptin synthesis (7). Serum leptin concentrations have been found to be higher in chronically insulin-treated diabetic children and adults with type 1 or 2 diabetes than in healthy controls and diabetics receiving other therapies (8). Furthermore, the leptin level has been reported to be lower before the start of insulin treatment in patients newly diagnosed with diabetes than in healthy controls (9, 10). According to recent reports, leptin may be present in high concentrations in some patients, but it is biologically inactive, so it cannot bind to its receptor (11, 12).

Therefore, the aim of the present study was to evaluate the status of serum leptin and biologically active leptin (bioLEP - functional leptin) levels in children with three forms of diabetes (newly diagnosed, well and poorly controlled) and healthy controls.

## 2 Materials and methods

### 2.1 Study subjects

The study included 94 children at the age of 11.53 ± 4.37 (58 boys and 36 girls). In a group of children with diabetes, 40 patients had newly diagnosed type 1 diabetes, aged 9.47 ± 3.91 years, 40 with long-term type 1 diabetes aged 12.61 ± 3.76 (long term defined as lasting more than one year). In the group of patients with long-term type 1 diabetes, 20 children had good metabolic control (aged 11.86 ± 3.94) and 20 with poor metabolic control aged 13.37 ± 3.5 years. All the parents of the children were Caucasian with no family history of type 1 or any type of diabetes. The control group consisted of 14 healthy children aged 14.33 ± 4.84 years, 9 girls and 5 boys. The diagnosis of type 1 diabetes was based on the criteria of the International Society of Pediatric and Adolescent Diabetes (13). Participants were recruited from the Department of Pediatrics, Pediatric Endocrinology and Diabetology, and the Outpatient Endocrinology Clinic between January 2019 and April 2021. Leptin and bioLEP levels were determined on the fifth day of hospitalization after stabilization of the patient’s general condition. The duration of diabetes treatment in the remaining patients was longer than 1 year. Patients with type 1 diabetes were treated with insulin analogues by intensive insulin therapy using pen or continuous subcutaneous insulin infusion with a personal insulin pump. Poor metabolic control of the disease was defined as a level of glycated hemoglobin (HbA1c) above 7%. At the same time, 14 healthy children were included in the control group. Type 1 diabetes was excluded from the control group based on medical history, clinical examination, biochemical (c-peptide and HbA1c) and immunological tests. Children with a body mass index, defined as BMI > 85^th^ percentile for sex and age, were also included in the study in both the diabetic and healthy children. BMI above the 85^th^ percentile was not the reason for exclusion from the study groups. Next, we divided the patients according to BMI percentiles into three groups: 1) underweight – less than the third percentile, 2) healthy weight - third percentile to less than the 85^th^ percentile, and 3) overweight and obesity – 85^th^ and greater than the 85^th^ percentile.

### 2.2 Biochemical analyses

Venous peripheral blood was drawn to a clotting activator tube (Sarstedt, Inc., Germany). Clotted samples were centrifuged for 10 minutes in 1000xg, at 4°C in a Centrifuge 5702 R (Eppendorf AG, Germany). Serum was transferred to 0.2 ml tubes (Greiner-bio-one, Austria) and cryopreserved at -80°C until the time of the experiment. Other clinical parameters were obtained from patient clinical records.

### 2.3 Leptin and bioLEP assay

Quantitative determination of total leptin was made using a sandwich enzyme-linked immunosorbent assay (ELISA) (E077, Mediagnost, Germany). BioLEP was also quantified by ELISA (L07, Mediagnost, Germany), however the analyte was captured by recombinant produced leptin receptor (SOB-R) immobilized on a microtiter plate. The entire procedure was prepared according to the manufacturer’s protocol. According to the manufactures specifications, the inter- and intra-assay coefficients of variation are below 10% for both ELISAs. Absorptiometric measurements were performed on a Tecan Infinite 200 PRO multimode reader (Tecan Group Ltd.; Männedorf, Switzerland). The quotients of biologically active leptin and leptin (bioLEP/LEP) were calculated.

### 2.4 Statistical analysis

All statistical analyses were performed using the STATISTICA software package (version 13.3, StatSoft Inc. 2017, Tulsa, OK, USA). Data were expressed as mean and SD, as well as range. Most variables did not follow a normal distribution, which was validated using the Shapiro-Wilk test, due to the nonparametric tests that were applied. Kruskal-Wallis ANOVA was used for multiple comparisons. A p-value below 0.05 was considered statistically significant.

### 2.5 Ethical approval

The study protocol was approved by the Bioethics Committee of the University of Rzeszow (Poland) 2018/03/08. All procedures performed in studies involving human participants were in accordance with the ethical standards of the institutional and/or national research committee and with the Declaration of Helsinki of 1964 and its subsequent amendments or comparable ethical standards. Written informed consent was obtained from legal guardians and/or children.

## 3 Results

The characteristics of the study group are presented in **Table 1**. Children with newly diagnosed diabetes (NDM1) were statistically younger than children with long-term diabetes, metabolically poorly controlled (DM1n) (p=0.007) and healthy children (p<0.001). We did not find statistical differences between the mean age of girls and boys in the study group. Statistically, diabetes was treated longer in children in the DM1n group (p<0.001). Children with newly diagnosed diabetes had statistically lower body weight than children in the DM1n (p<0.001) and control groups (p=0.006). Children with newly diagnosed diabetes had a statistically lower BMI than children in the DM1n group (p<0.001) and healthy children (p=0.002). Moreover, children in the NDM1 group had a statistically higher level of HbA1c compared to children with long-term diabetes, metabolically well controlled (DM1w) (p<0.001) group and healthy children (p<0.001). Children with long-term and well-controlled diabetes had statistically lower HbA1c levels than children in the DM1n group (p<0.001). There were no statistical differences in cholesterol and LDL levels. We observed a statistical difference in HDL level between children from the NDM1 group and DM1w (p=0.036). Finally, children in the NDM1 group had statistically higher triglyceride levels than children in the DM1w (p<0.001), DM1n (p=0.002) group and healthy children (p=0.02).

**Table 1:** General characteristics of children included in the study.*.

		NDM1	DM1		Healthy controls	p
			DM1w	DM1n		
Sex (F/M)		11/29	6/14	10/10	5/9	
Age (years)	mean ± SD	9.47 ± 3.91	11.86 ± 3.94	13.37 ± 3.5	14.33 ± 4.84	0.001
	range	2.2 - 16.1	2.9 - 17.9	5.1 - 17.8	3.6 - 17.6	
Duration of disease (months)	mean ± SD	-	27 ± 10.8	79 ± 31.9	-	<0.001
	range	-	11 - 49	27 - 152	-	
Weight (kg)	mean ± SD	34.64 ± 16.4	43 ± 16.38	55.44 ± 17.4	54.9 ± 18.7	0.001
	range	12 - 72	13.5 - 72	20.5 - 83	15 - 87	
BMI (kg/m^2^)	mean ± SD	16.84 ± 3.67	18.05 ± 2.69	21.64 ± 3.54	21.22 ± 3.05	<0.001
	range	12.5 - 26.34	14.31 - 24.16	16.27 - 28.06	15.03 - 26.34	
BMI percentiles						
<3^rd^	n (%)	7 (17.5)	0	0	0	
3^rd^ – 85^th^	n (%)	27 (67.5)	16 (80)	13 (65)	11 (79)	
<85^tt^	n (%)	6 (15)	4 (20)	7 (35)	3 (21)	
HbA1c (%)	mean ± SD	11.78 ± 2.58	6.47 ± 0.48	10.18 ± 2.18	5.29 ± 0.19	<0.001
	range	6.93 - 17.03	5.23 - 6.9	8 - 15.1	4.9 - 5.6	
TC (mg%)	mean ± SD	178.23 ± 55.66	170.5 ± 27.95	179 ± 47.42	176.07 ± 11.81	0.929
	range	16 - 297	129 - 224	125 - 292	150 - 190	
HDL (mg%)	mean ± SD	54.38 ± 30.13	62.65 ± 17.9	58.45 ± 9.29	49.93 ± 7.67	0.007
	range	19 - 181	29 - 97	40 - 78	40 - 68	
LDL (mg%)	mean ± SD	95.38 ± 44.1	92.1 ± 20.96	99 ± 35.52	82.14 ± 17.65	0.543
	range	30 - 206	45 - 124	44 - 182	60 - 115	
TG (mg%)	mean ± SD	297.93 ± 319.63	62.65 ± 17.9	112.7 ± 72.24	99.86 ± 27.72	<0.001
	range	53 - 1800	34 - 139	41 - 328	58 - 156	

*NDM1, patients with newly diagnosed diabetes; DM1w, patients with long-term diabetes who are metabolically well-controlled; DM1n, patients with long-term diabetes; metabolically poorly controlled; BMI, body mass index; HbA1c, glycated hemoglobin A1; TC, cholesterol; TG, triglycerides. Data are expressed as mean ± SD.

**Figure 1:**
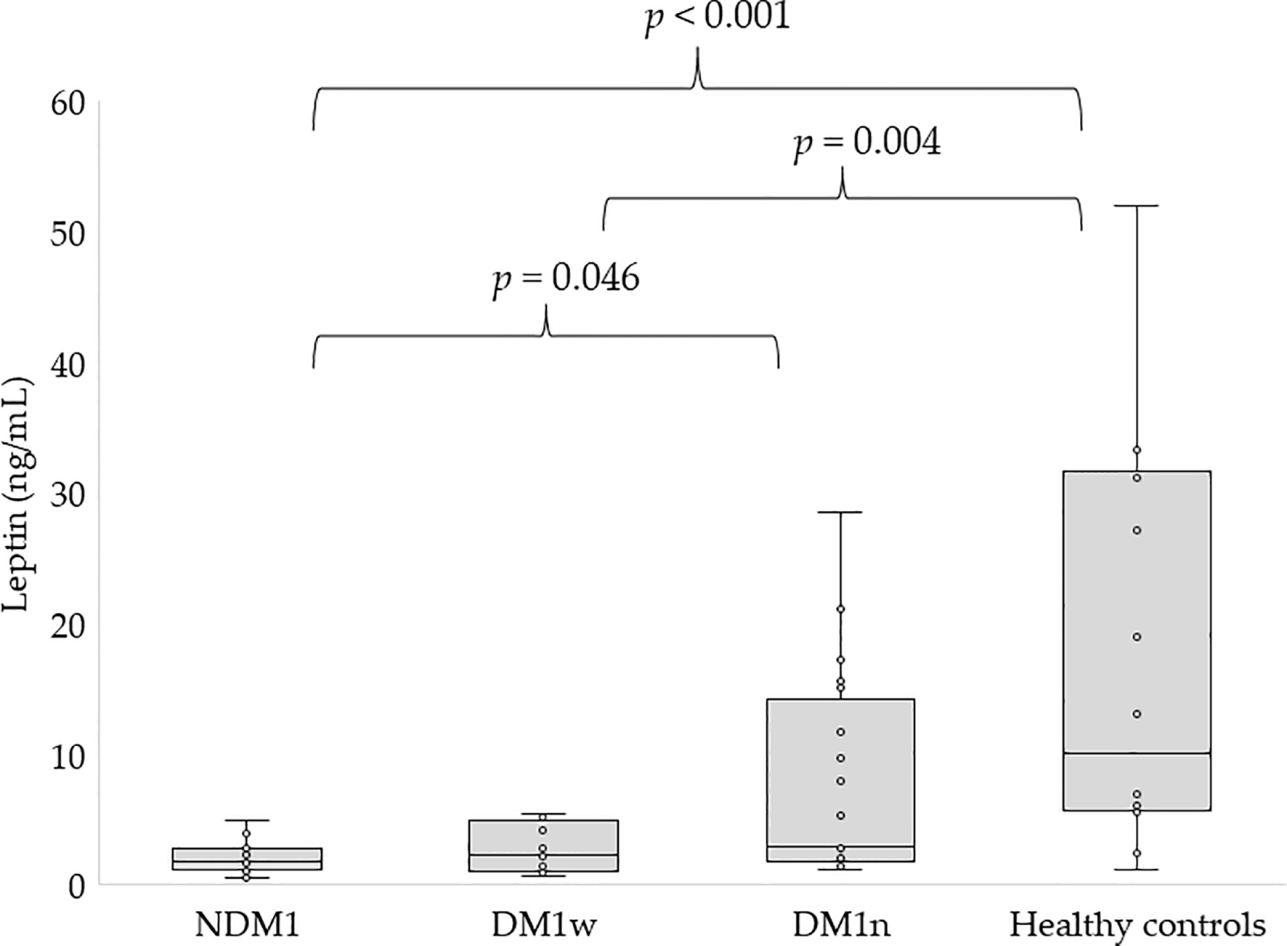
Concentration of leptin in studied groups, NDM1, patients with newly diagnosed diabetes; DM1w, patients with long-term diabetes who are metabolically well-controlled; DM1n, patients with long-term diabetes; metabolically poorly controlled.

**Figure 2:**
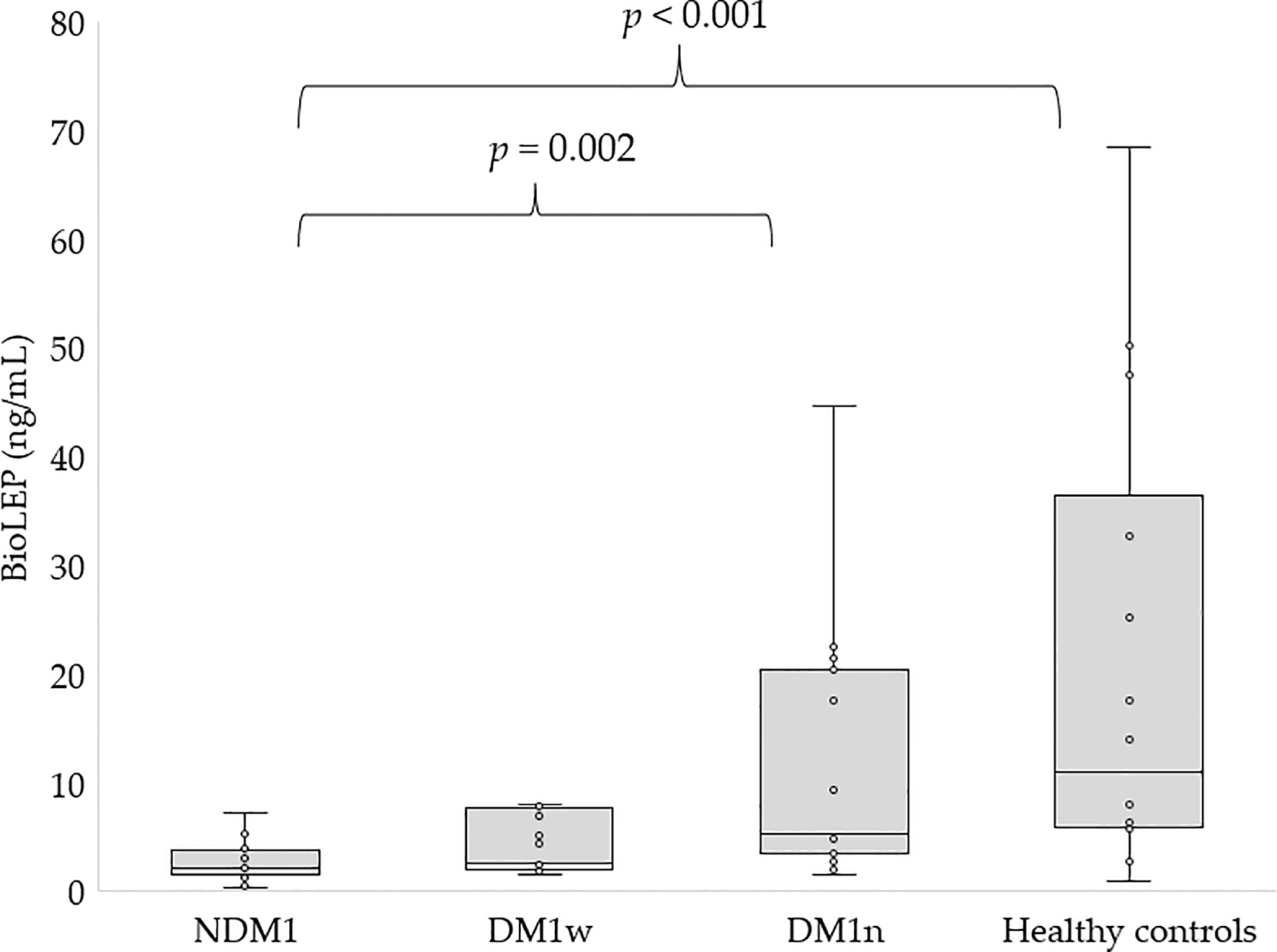
Concentration of bioLEP in studied groups, NDM1, patients with newly diagnosed diabetes; DM1w, patients with long-term diabetes who are metabolically well-controlled; DM1n, patients with long-term diabetes; metabolically poorly controlled.

**Figures 1**, **2** present the level of leptin and bioLEP in the studied groups. We found a statistically higher concentration of leptin in the group of children with newly diagnosed diabetes compared to the children in the DM1n group (11.19 ± 52.16 vs 7.75 ± 7.94 ng/mL, p=0.046) and lower than in healthy children (11.19 ± 52.16 vs 20.94 ± 23.29 ng/mL, p<0.001). Furthermore, children in the DM1w group had statistically lower levels of this hormone than healthy children (5.11 ± 8.59 vs 20.94 ± 23.29 ng/mL, p=0.004, **Figure 1**). The level of bioLEP was statistically increased in children with NMD1 compared to children in the DM1n group (17.2 ± 83.66 vs 11.33 ± 11.03 ng/mL, p=0.002) and lower than in healthy children (17.2 ± 83.66 vs 20.86 ± 21.16 ng/mL, p<0.001). The bioLEP level in the participants in the DM1w group was 7.84 ± 12.45 ng/ml and did not differ statistically from other study groups.

Individual bioLEP/LEP quotients in children with NDM1 were significantly decreased compared to children in the DM1w and DM1n group (1.28 ± 0.52 vs. 1.72 ± 0.43, p<0.05 and 1.63 ± 0.38, p<0.05, respectively). The ratio of bioLEP to leptin in the group of healthy children was close to 1 (1.03 ± 0.19) and was statistically lower than in the DM1w and DM1n groups (p<0.001).

**Table 2** presents differences in leptin, bioLEP, and bioLEP/leptin levels depending on the sex of the study participants. There were no statistical differences in the levels of leptin, bioLEP, and bioLEP/leptin among children with NDM1. Both in the DM1w and DM1n group, leptin and bioLEP were higher in girls than in boys. There was no difference in bioLEP/leptin in either group. Surprisingly, leptin and bioLEP levels were higher in healthy boys than in girls. Individual quotients of bioLEP/LEP in healthy boys and girls were close to 1.

**Table 2:** Leptin parameters depending on the sex of the participants.*.

		Leptin, ng/mL	BioLEP, ng/mL	BioLEP/Leptin
**NDM1**	Boys	14.28 ± 61.22	3.99 ± 5.86	1.18 ± 0.43
	Girls	3.06 ± 3.59	52.03 ± 159.25	1.55 ± 0.67
	*p*	0.906	0.431	0.134
**DM1w**	Boys	2.07 ± 1.33	3.29 ± 2	1.69 ± 0.44
	Girls	12.21 ± 13.75	18.45 ± 19.64	1.78 ± 0.53
	*p*	0.063	0.029	0.773
**DM1n**	Boys	2.76 ± 2.04	4.19 ± 2.28	1.63 ± 0.44
	Girls	11.83 ± 8.71	17.18 ± 11.97	1.62 ± 0.33
	*p*	0.019	0.008	0.879
**Healthy controls**	Boys	34.03 ± 34.77	33.99 ± 30.44	1.05 ± 0.34
	Girls	13.67 ± 10.5	13.56 ± 9.84	1.02 ± 0.07
	*p*	0.689	0.594	0.505

*NDM1, patients with newly diagnosed diabetes; DM1w, patients with long-term diabetes who are metabolically well-controlled; DM1n, patients with long-term diabetes; metabolically poorly controlled.

**Tables 3**, **4** present the level of leptin and bioLEP depending on the BMI percentiles. Children below the third percentile were only in the NDM1 group. Among children of normal weight, leptin levels were significantly different between healthy children and children with newly diagnosed diabetes, as well as participants from the DM1w group. We did not observe any difference in leptin levels between the other groups of children with a BMI between the third and 85^th^ percentiles, and between children above the 85^th^ percentile. Among children with newly diagnosed diabetes, leptin levels were significantly different between underweight, overweight, and obese children (p<0.001). Moreover, children of normal weight had statistically lower leptin levels than children with a BMI above the 85^th^ percentile (p=0.003). In the group of children with DM1w and DM1n, there were no differences in leptin levels between children with normal weight and children with a BMI greater than the 85th percentile (p=0.122 and p=0.096, respectively). Moreover, there were no differences in the level of this hormone between the groups in the control group (p=0.291).

**Table 3:** Concentration of leptin depending on the BMI percentile.*.

BMI	NDM1	DM1w	DM1n	HC	*p*	*p* NDM1/HC	*p* DM1w/HC	*p* DM1n/HC	*p* NDM1/ DM1w	*p* NDM1/ DM1n	*p* DM1w/ DM1n
<3^rd^	1.21 ± 0.43	-	-	-	-	-	-	-	-	-	-
3-85^th^	2.25 ± 2.38	3.41 ± 4.28	5.24 ± 5.58	18.33 ± 23.74	<0.001	<0.001	0.005	0.459	0.999	0.253	0.716
>85^th^	63.09 ± 131.85	11.94 ± 17.26	12.42 ± 9.93	30.48 ± 23.08	0.479	0.999	0.755	0.999	0.999	0.999	0.999

*NDM1, patients with newly diagnosed diabetes; DM1w, patients with long-term diabetes who are metabolically well-controlled; DM1n, patients with long-term diabetes; metabolically poorly controlled; HC, healthy controls.

**Table 4:** Concentration of bioLEP depending on the BMI percentile.*.

BMI	NDM1	DM1w	DM1n	HC	*p*	*p* NDM1/HC	*p* DM1w/HC	*p* DM1n/HC	*p* NDM1/ DM1w	*p* NDM1/ DM1n	*p* DM1w/ DM1n
<3^rd^	1.15 ± 0.53	-	-	-	-	-	-	-	-	-	-
3-85^th^	22.42 ± 101.88	5.46 ± 6.46	8.04 ± 7.51	18.66 ± 21.7	<0.001	0.002	0.151	0.999	0.999	0.031	0.983
>85^th^	63.09 ± 131.85	17.34 ± 24.95	17.45 ± 14.32	28.92 ± 20.8	0.63	0.999	0.999	0.999	0.999	0.999	0.999

*NDM1, patients with newly diagnosed diabetes; DM1w, patients with long-term diabetes who are metabolically well-controlled; DM1n, patients with long-term diabetes; metabolically poorly controlled; HC, healthy controls.

The level of bioLEP was significantly different between children in the NDM1 group and healthy children and children in the DM1n group. In the NDM1 group, the level of bioLEP was significantly higher in children with normal weight and overweight and obese than in the group of underweight children (p=0.037 and p<0.001, respectively). Moreover, children with a BMI between the 3^rd^ and 85^th^ percentiles had significantly lower bioLEP levels than overweight and obese children (p=0.013).

In the group of children with DM1w and DM1n, there was no difference in bioLEP levels between children with normal weight and children with a BMI above the 85th percentile (p=0.177 and p=0.157, respectively). In addition, there was no difference in the level of this hormone between the groups in the control group (p=0.368).

The dependence between leptin and bioLEP level and patient general characteristics was estimated using Spearman’s correlation. Spearman rank correlation coefficients and p values for each separate disease are presented in **Tables 5**, **6**. We found a strong positive correlation between leptin level and weight and BMI in children with NDM1 and healthy controls (R=0.524, p<0.001; R=0.7, p<0.001 and R=0.59, p=0.025; R=0.6, p=0.023, respectively). In addition, in the group of children with DM1n there was a moderate and positive correlation between leptin and total cholesterol and LDL level. No other association was demonstrated between leptin concentration and other parameters analyzed.

**Table 5:** Spearman’s rank correlation coefficients and p values for leptin.*.

	NMD1		DM1w		DM1n		HC	
	*R*	*p*	*R*	*p*	*R*	*p*	*R*	*p*
Age	0.291	0.068	-0.014	0.952	0.099	0.678	-0.120	0.684
Weight	0.524	<0.001	0.113	0.636	0.146	0.537	0.594	0.025
BMI	0.7	<0.001	0.26	0.268	0.444	0.05	0.6	0.023
Hb1Ac	0.163	0.315	0.226	0.338	0.074	0.757	0.083	0.778
TC	-0.0437	0.788	0.195	0.409	0.47	0.036	-0.212	0.468
HDL	-0.0166	0.919	0.142	0.55	0.33	0.155	0.528	0.053
LDL	0.223	0.166	0.093	0.696	0.517	0.02	-0.498	0.07
TG	-0.001	0.998	0.049	0.838	0.411	0.072	0.311	0.28

*NDM1, patients with newly diagnosed diabetes; DM1w, patients with long-term diabetes who are metabolically well-controlled; DM1n, patients with long-term diabetes; metabolically poorly controlled; HC, healthy controls.

**Table 6:** Spearman’s rank correlation coefficients and p values for bioLEP.*.

	NMD1		DM1w		DM1n		HC	
	*R*	*p*	*R*	*p*	*R*	*p*	*R*	*p*
Age	0.230	0.154	-0.13	0.585	0.137	0.563	-0.183	0.531
Weight	0.487	<0.001	-0.03	0.902	0.155	0.512	0.491	0.075
BMI	0.706	<0.001	0.107	0.654	0.490	0.028	0.459	0.098
Hb1Ac	0.181	0.263	0.262	0.265	0.109	0.646	0.110	0.708
TC	-0.034	0.834	0.277	0.238	0.492	0.028	-0.331	0.248
HDL	-0.096	0.558	0.078	0.743	0.325	0.162	0.559	0.038
LDL	0.214	0.186	0.275	0.240	0.577	0.008	-0.54	0.046
TG	0.038	0.817	0.058	0.808	0.495	0.027	0.278	0.337

*NDM1, patients with newly diagnosed diabetes; DM1w, patients with long-term diabetes who are metabolically well-controlled; DM1n, patients with long-term diabetes; metabolically poorly controlled; HC, healthy controls.

Regarding bioLEP, we observed a strong positive correlation between bioLEP level and weight and BMI in children with newly diagnosed diabetes (R=0.487, p<0.001 and R=0.706, p<0.001). Furthermore, the association of bioLEP with BMI, total cholesterol, LGL, and triglycerides had a moderate and increasing trend in children with DM1n (Tab. 5). BioLEP levels were positively correlated with HDL (R=0.559, p=0.038) and negatively correlated with LDL (R=-0.54, p=0.046) in healthy children.

We did not find any additional associations between other analyzed parameters. The level of leptin and bioLEP strongly correlated with each other in all study groups (**Table 7**).

**Table 7:** Spearman’s rank correlation coefficients and p values for leptin and bioLEP.

	NDM1	DMw	DMn	HC
**R**	0.876	0.895	0.948	0.982
***p***	p<0.0001	p<0.0001	p<0.0001	p<0.0001

## 4 Discussion

In the current study, leptin and bioLEP concentrations were assessed in children with type I diabetes and healthy children. Lower levels of leptin were found in children with diabetes compared to healthy children. Moreover, we found a statistically higher concentration of leptin in the group of children with newly diagnosed diabetes compared to children in the diabetic group with poor metabolic control and a decrease than in healthy children. Moreover, children from the metabolically well-controlled group had statistically lower levels of this hormone than healthy children. Hanaki et al. showed lower levels of leptin in newly diagnosed children with DM1 compared to healthy children (3.3 ± 0.2 vs 6.2 ± 0.9 ng/mL; p<0.005), and its level increased with the use of insulin treatment (14). Similarly, serum from newly diagnosed children with diabetes had significantly lower levels of leptin (mean 1.28 ± 1.60 ng/ml) compared to healthy children (mean 2.2 ng/ml) in the study by Kiess et al. (10). Contrary to our results, Morales et al. showed higher serum leptin levels in children with DM1 compared to healthy children (5.1 vs 2.7 ng/mL) (15). Insulin treatment increases leptin levels (5.18 ± 5.48 ng/ml) in children with newly diagnosed diabetes, which is consistent with our results (10). However, among our patients, leptin levels in children with well-controlled DM1 were significantly lower than in healthy controls (5.11 ± 8.59 vs 20.94 ± 23.29 ng/mL). Leptin plays an essential role in maintaining body weight and glucose homeostasis (16). This is done by its central and peripheral actions. There is a direct relationship between leptin and insulin. The presence of leptin receptors in pancreatic beta cells indicates the involvement of leptin in the pancreatic endocrine system, including the regulation of insulin secretion by beta cells. It is assumed that insulin increases the production of leptin by adipose tissue, while leptin inhibits insulin secretion and insulin gene expression. The repressive effect of leptin on insulin production is regulated both by the autonomic nervous system and directly by affecting leptin receptors in beta cells (17). Leptin can inhibit basal and glucose-stimulated insulin secretion (18). This occurs through several mechanisms, including the activation of ATP-dependent potassium channels resulting in membrane hyperpolarization and suppression of insulin secretion (19–21). The above-mentioned relationships may explain the decreased level of leptin in patients with insulin deficiency during the course of type 1 diabetes.

In addition, the present study showed a significant correlation between leptin concentration and BMI. Among children with newly diagnosed DM1, leptin concentrations differed significantly between underweight, overweight, and obese children. Additionally, in the NDM1 group, children with normal body weight had statistically lower leptin levels than children with BMI above the 85^th^ percentile. In the group of children with DM1w and DM1n, there were no differences in leptin concentration between children with normal weight and children with BMI above the 85^th^ percentile. In addition, there was no difference in the level of this hormone between the groups according to BMI in the control group. In studies by Soliman et al., higher levels of leptin were observed in children with a higher BMI (22). Kiess et al. also found a positive correlation between leptin and BMI (R=0.42, p<0.0001) (10). In overweight patients with DM1, leptin showed a significant positive correlation with hip circumference and BMI (23).

As in the studies by Morales et al. and Kiess et al., no correlation were found between leptin concentration and HbA1c level and lipid metabolism parameters in children with NDM1 (10, 15). In the group of children with DM1n, a moderate and positive correlation was found between leptin level and total cholesterol and LDL was found. In the conducted studies, similarly to the study by Kratzsch et al., no significant differences were found in terms of leptin concentration and severity of acid-base disturbances (24).

Recent studies have shown that mutations in the leptin gene can lead to leptin dysfunction. Congenital leptin deficiency is characterized by excessive appetite and severe early obesity, as well as metabolic and endocrine disorders. The disease is caused by mutations in the leptin gene, which usually lead to defects in leptin synthesis, and thus to the absence or very low levels of this hormone in the circulation (25). Functional leptin deficiency is characterized by high levels of circulating immunoreactive leptin, but decreased hormone bioactivity due to defective receptor binding. Mutations in the leptin gene have been described in obese patients in whom circulating immunoreactive leptin levels were detectable while bioLep levels were low. The use of recombinant human leptin led to a rapid improvement in eating behavior and weight loss (11, 26).

To our knowledge, this is the first study to describe bioLEP levels among children with DM1 with different forms of disease control. In our study, the level of bioLEP differed significantly between the children of the NDM1 group (17.2 ± 83.66, range: 0.33-532 ng/mL) and the children of the DM1n group (11.33 ± 11.03, range: 1.58-44.63 ng/mL) and healthy controls (20.86 ± 21.16, range: 0.99-68.47 ng/mL). Moreover, children with NDM1 compared to children with DM1n have lower levels of leptin but higher levels of bioLEP. In the NDM1 group, the level of bioLEP was significantly higher in children with normal weight and overweight and obese than in the group of underweight children. Furthermore, children with a BMI between the 3^rd^ and 85^th^ percentiles had significantly lower bioLEP levels than overweight and obese children. In the group of children with DM1w and DM1n, there were no differences in bioLEP levels between children with normal weight and children with BMI greater than the 85^th^ percentile. Additionally, there were no differences in the level of this hormone between the groups in the control group. BioLEP was correlated with BMI among children with NDM1 and DM1N. This correlation was at a level comparable to the correlation between total LEP and BMI. Furthermore, the hormone was positively correlated with LDL and glycerides among children with poor diabetes control. Interestingly, bioLEP was positively correlated with HDL and negatively with LDL among healthy children. BioLEP levels were positively correlated with age and BMI among children with severe early-onset obesity (12). The better concentration of bioLEP than total leptin might reflect the amount of hormone that is capable of exerting a biological effect (27). Niklowitz et al. found that bioLEP levels did not differ between prepubertal girls and boys, while we observed a trend for higher bioLEP levels in girls compared to boys with DM1w and DM1n (27). Similar observations have recently been made among obese children (12).

Insulin deficiency in type 1 diabetes results in a state of increased lipolysis of adipocytes, which causes an increase in circulating free fatty acids and ultimately ketonemia. Both of these metabolites can reduce the ability of adipocytes to secrete leptin, signaling an “energy deficit.” Therefore, type 1 diabetes is a condition in which one can speak of a “relative leptin deficiency” (28). Experimental studies and clinical observations indicate a relationship between insulin and leptin release (29). Despite many studies published in recent years, many aspects of leptin secretion, action, and mechanisms of its influence on carbohydrate and fat metabolism are still to be clarified. In our opinion, studies evaluating the status of bioLEP in diabetes may also contribute to a better understanding of the mechanisms that regulate metabolism. Many studies have suggested that leptin can be used as an antidiabetic drug or in addition to insulin therapy in patients with insulin-dependent diabetes (30–33). The results of experimental studies are encouraging; however, the use of such a therapy in humans requires further clinical trials.

## 5 Conclusion and limitation

Although our study provides new information on leptin and bioLEP among pediatric patients with varying degrees of DM1 control, several limitations of the study should be mentioned. First, this study is a single-center study with a small group of children. The COVID-19 pandemic prevented us from collecting a larger group of patients. Second, we did not analyze the soluble leptin receptor in the serum of the participants. Therefore, further research is needed containing larger groups of patients differing in sex, age, and degree of sexual maturation.

## Data availability statement

The original contributions presented in the study are included in the article/supplementary material. Further inquiries can be directed to the corresponding author.

## Ethics statement

The studies involving human participants were reviewed and approved by Bioethics Committee of the University of Rzeszow (Poland) 2018/03/08. Written informed consent to participate in this study was provided by the participants’ legal guardian/next of kin.

## Author contributions

Conceptualization: KJ-K, JT and AM. Data curation: KJ-K. Formal analysis: SG. Investigation: KJ-K, KŁ, IK, AL and JT. Methodology: KJ-K. Project administration: KJ-K and AM. Resources: KJ-K. Software: SG. Supervision: AM. Writing-original draft: KJ-K and SG. Writing-review and editing: KJ-K, SG and AM. All authors contributed to the article and approved the submitted version.
